# Prevalence and Molecular Identification of *Schistosoma haematobium* among Children in Lusaka and Siavonga Districts, Zambia

**DOI:** 10.3390/tropicalmed7090239

**Published:** 2022-09-10

**Authors:** Rabecca Tembo, Walter Muleya, John Yabe, Henson Kainga, King S. Nalubamba, Mildred Zulu, Florence Mwaba, Shereen Ahmed Saad, Moses Kamwela, Andrew N. Mukubesa, Ngula Monde, Simegnew Adugna Kallu, Natalia Mbewe, Andrew M. Phiri

**Affiliations:** 1 Department of Pathology and Microbiology, School of Medicine, University of Zambia, Lusaka P.O. Box 50110, Zambia; 2 Department of Clinical Sciences, School of Veterinary Medicine, University of Zambia, Lusaka P.O. Box 32379, Zambia; 3 Africa Center of Excellence for Infectious Diseases of Humans, University of Zambia, Lusaka P.O Box 32379, Zambia; 4 Department of Biomedical Sciences, School of Veterinary Medicine, University of Zambia, Lusaka P.O. Box 32379, Zambia; 5 Department of Para Clinical Studies, School of Veterinary Medicine, University of Zambia, Lusaka P.O. Box 32379, Zambia; 6 School of Veterinary Medicine, University of Namibia, P.O. Box 13301, Windhoek 1005, Namibia; 7 Department of Veterinary Epidemiology and Public Health, Faculty of Veterinary Medicine, Lilongwe University of Agriculture and Natural Resources, Lilongwe 207203, Malawi or; 8 Department of Disease Control, School of Veterinary Medicine, Zambia Animals, University of Zambia, Lusaka P.O. Box 32379, Zambia; 9 Department of Clinical Studies, College of Veterinary Science, University of Bahr El Ghazal, Wau P.O. Box 10739, South Sudan; 10 Department of Pharmacology, Faculty of Pharmacy, Lusaka Apex Medical University, Lusaka P.O. Box 31909, Zambia; 11 Department of Biomedical Sciences, Tropical Diseases Research Centre, Ndola P.O. Box 71769, Zambia; 12 College of Veterinary Medicine, Haramaya University, Dire Dawa P.O. Box 138, Ethiopia; 13 Department of Basic and Clinical Nursing Sciences, School of Nursing Sciences, University of Zambia, Lusaka P.O. Box 50110, Zambia

**Keywords:** *Schistosoma*, schistosomiasis, urinary schistosomiasis, *Schistosoma haematobium*

## Abstract

Schistosomiasis remains a public health concern in Zambia. Urinary schistosomiasis caused by *Schistosoma haematobium* is the most widely distributed infection. The aim of the current study was to determine the prevalence and risk factors of urinary schistosomiasis and identify the strain of *S. haematobium* among children in the Siavonga and Lusaka districts in Zambia. Urine samples were collected from 421 primary school children and *S. haematobium* eggs were examined under light microscopy. A semi-structured questionnaire was used to obtain information on the socio-demographic characteristics and the potential risk factors for urinary schistosomiasis. DNA of the parasite eggs was extracted from urine samples and the internal transcribed spacer gene was amplified, sequenced and phylogenetically analysed. The overall prevalence of *S. haematobium* was 9.7% (41/421) (95% CI: 7.16–13.08), male participants made up 6.2% (26/232) (95% CI: 4.15–9.03), having a higher burden of disease than female participants who made up 3.5% (15/421) (95% CI: 2.01–5.94). The age group of 11–15 years had the highest overall prevalence of 8.3% (35/421) (5.94–11.48). Participants that did not go fishing were 0.008 times less likely to be positive for schistosomiasis while participants whose urine was blood-tinged or cloudy on physical examination and those that lived close to water bodies were 9.98 and 11.66 times more likely to test positive for schistosomiasis, respectively. A phylogenetic tree analysis indicated that *S. haematobium* isolates were closely related to pure *S. haematobium* from Zimbabwe and hybrids of *S. haematobium* × *S. bovis* from Benin, Senegal and Malawi. The current study shows that urinary schistosomiasis is endemic in the study areas and is associated with water contact, and *S. haematobium* isolated is closely related to hybrids of *S. bovis* × *S. haematobium* strain, indicating the zoonotic potential of this parasite.

## 1. Introduction

Schistosomiasis is an important neglected tropical disease and is second to malaria in terms of parasite-induced morbidity and mortality [1]. It occurs in many developing countries in tropical and sub-tropical Africa, Middle East, Asia and Latin America. The disease accounts for up to 90% of cases in sub-Saharan Africa in a population that accounts for 13% of the world’s population [2].

The three main species that cause human schistosomiasis are *Schistosoma haematobium*, which causes urinary schistosomiasis, *S. mansoni* and *S. japonicum*, which cause intestinal schistosomiasis. Other species such as *S. guineensis* and *S. intercalatum* cause intestinal schistosomiasis, but are less prevalent [2,3]. Infection occurs when people come in to contact with fresh water bodies infested with cercariae released by specific intermediate host snails which have been infected by miracidia released from the eggs of adult worms [2,4]. The highest burden of the disease is seen in children, especially in the ages between 10–15 years [5]. Children harbouring these infections usually remain asymptomatic and may only show complications such as lesions of the bladder as adult worms and eggs migrate through the body. Even though infection due to *S. haematobium* is rarely fatal, disabling morbidities such as anaemia and impaired cognitive development have been observed in children [5].

In Zambia, schistosomiasis due to *S. haematobium* and *S. mansoni* is prevalent in all the 10 provinces. The most common form is urinary schistosomiasis caused by *S. haematobium* [6]. Two million people out of the population of about thirteen million are said to be infected, while three million are living under the threat of being infected [7]. It is especially prevalent in rural districts close to the lakes and rivers [4]. However, urban foci have also been identified in many endemic areas due to development of peri-urban squatter compounds. These tend to lack an established water supply infrastructure and sometimes a small number of year-round streams associated with high infection rates run through these settlements [8,9]. Schistosomiasis remains highly prevalent in Zambia despite efforts to control it [6]. Mass drug administration using praziquantel has been the main disease control strategy. However, reinfections have been reported especially as people revert to water contact activities even after being treated successfully with praziquantel [6,10,11].

Most epidemiological assessments on the burden of urinary schistosomiasis have relied on microscopy, providing a cheap way for the detection of schistosome eggs in urine in many endemic countries [12,13]. In Zambia, for example, most prevalence studies conducted have been based on microscopic results [6]. Diagnosis is based on the detection of parasite eggs in the urine and differentiating the parasite based on egg morphology. However, this method is associated with low sensitivity and is also highly unspecific because it cannot characterize the parasites into species and sub-species [14,15]. Molecular based tools such as the use of nuclear and mitochondrial genes have proved to be efficient tools for the detection and identification of *Schistosoma* [14].

Molecular techniques based on the nuclear internal transcribed spacer region (ITS) can be used to type strains which also allows for the detection of hybrids between animal and human schistosomes [13]. The current study, therefore, set out to investigate the prevalence of urinary schistosomiasis among school children in the Lusaka and Siavonga districts in Zambia using microscopy, and to identify the *S. haematobium* strain detected on microscopy using molecular techniques based on the nuclear ITS gene. This knowledge will provide new insights for understanding the evolution and epidemiology of urinary schistosomiasis in Zambia. While only one study has detected *S. haematobium* in urine samples using polymerase chain reaction (PCR), to the best of our knowledge, this is the first study that has sequenced and identified the strain of *S. haematobium* based on molecular techniques and phylogenetic tree analysis in Zambia.

## 2. Materials and Methods

### 2.1. Study Site and Design

**Figure 1:**
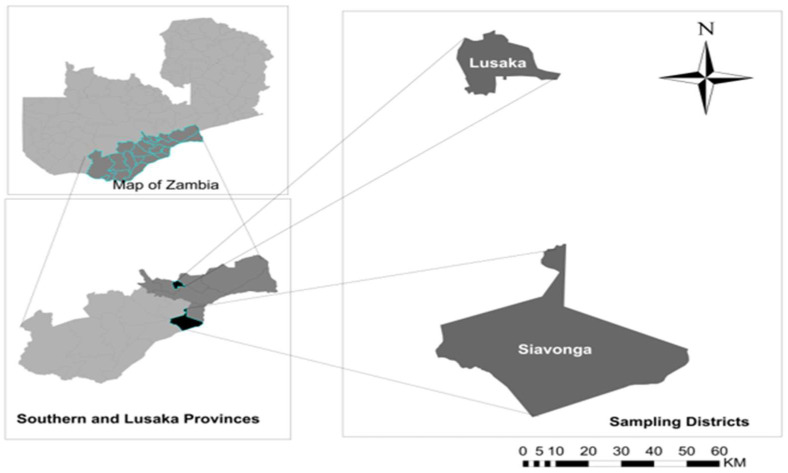
Map showing the two study areas, Siavonga and Lusaka districts.

We conducted a cross-sectional study in selected schools in the Siavonga (Southern Province) and Lusaka (Lusaka province) districts of Zambia (Figure 1). The sites were chosen based on the previous reports of high schistosomiasis disease burden [16,17]. Further, Siavonga is a rural area on the shore of the Lake Kariba and Zambezi River while Lusaka is an urban area. Samples were collected from schools in both districts. Children in Siavonga are mostly involved in water contact activities such as swimming, bathing and fetching water among others, while those in Lusaka are not.

### 2.2. Sample Collection

Two hundred and twenty urine samples were obtained from the primary schools in Siavonga, namely Siamatika (*n* = 42), Butete (*n* = 41), Nabutezi (*n* = 47), Mpango (*n* = 44) and Chininde (*n* = 46) while two hundred and one urine samples were obtained from Ng’ombe Primary School (*n* = 201) in Lusaka district. All the children from whom samples were obtained were randomly selected. We also obtained 20 clinical samples that were confirmed positive for *S. haematobium* from Siavonga District Hospital (*n* = 8) and Ng’ombe Clinic (*n* = 12) in Lusaka. These samples were obtained from children aged between 9 and15 years who presented with haematuria and dysuria. Single urine samples were collected from the children between 9 am and 14 pm and stored in clean containers in cooler boxes containing ice packs. Samples were transported to Siavonga District Hospital and Ng’ombe Clinic Laboratories for microscopic examination on the same day of collection. For each child, information such as age, sex, grade, knowledge about schistosomiasis, herding of livestock and involvement in activities such as swimming, bathing, playing, fishing, washing and fetching water was obtained using a semi-structured questionnaire administered to the children with the help of class teachers in the Chitonga and Chinyanja local languages.

### 2.3. Sample Preparation and Processing

#### 2.3.1. Microscopic Examination

The urine samples were processed using the previously described urine centrifugation method [14]. Briefly, 10 mL of urine was poured into a centrifuge tube and was spun at 15,000 rpm for 5 min. The supernatant was then discarded, and the sediments were obtained using a pasture pipette and 2 drops of the sediments were placed on a frosted glass slide and covered with a coverslip. The slides were then examined using a light microscope at ×10 and ×40 objective lenses for the presence of *S. haematobium* eggs. The eggs were identified and distinguished from other schistosomes by presence of a conspicuous lateral spine. The *S. haematobium* eggs were counted and infection intensity was recoded as the number of ova/10 mL of urine and categorized as light (≤50 ova/10 mL of urine) and heavy (≥50 ova/10 mL of urine) (5).

#### 2.3.2. DNA Extraction and Amplification

DNA was extracted directly from parasite eggs in urine samples using a commercial genomic DNA extraction kit, Qiagen QlAamp DNA miniprep kit (Qiagen Group, Germany) according to the manufacturer’s instructions. The extracted DNA was amplified using one set of primers namely ITTS2F (TAACAAGGTTTCCGTAGGTGAA) and ITTS1R (TGCTTAAGTTCAGCGGGT) targeting the ITS region of *S. haematobium* according to a previously described PCR protocol [18]. The expected amplicon band size was 981 bp. The PCR reaction volume was 20 µL and comprised of 5 ng/µL of the DNA template, 3 µL of nuclease-free water, 10 µL of One Taq quick load (Biolabs, Durham, North Carolina, USA) and 0.5 µM of each primer on a thermal cycler (Applied Biosystems, Chiba, Japan). The following PCR conditions were used: 95 °C for 5 min of initial denaturation followed by 40 cycles of 30 s at 95 °C, 30 s at 56 °C, 1 min at 72 °C and a final extension step of 7 min at 72 °C. Amplicons were then visualised on 1.5% agarose gel stained with ethidium bromide.

#### 2.3.3. Purification of the PCR Products and Cycle Sequencing

The amplified PCR products were purified using the Zymo Research Genomic DNA Clean and Concentrator^TM^-25 kit (Irvine, CA, USA) according to the manufacturer’s instructions. The purified DNA products were then subjected to sequencing using the brilliant dye terminator ver.3.1 (NimaGen, Nijmegen, The Netherlands) according to the manufacturer’s instructions. The sequence products were precipitated using the ethanol precipitation method followed by denaturation with formamide and capillary electrophoresis on the ABI 3500 Genetic Analyser (Applied Biosystems).

### 2.4. Data Analysis

#### 2.4.1. Epidemiological Data Analysis

Data were entered, cleaned and validated in a Microsoft™ excel spreadsheet (MS Office Excel^®^ 2016). Schistosomiasis test results (positive or negative) were the dependent variable. The data were then exported to SPSS software ver. 21 (IBM Corp., Armonk, NY, USA). A univariate analysis was conducted for descriptive results such as frequencies and proportions. An analysis of association for potential risk factors with schistosomiasis positivity was conducted using Pearson chi-square (and Fisher’s exact test where appropriate) in a bivariate analysis and further screening for multicollinearity was checked by variance inflation factors (VIF) (VIF value < 1.0) and tolerance (value > 0.2). Thereafter, a multivariable linear regression model was fitted, which included variables that retained significance (*p* < 0.250) at a univariate linear regression analysis. For the multivariate analysis, a stepwise regression method and forward algorithms were used, then independent variables with *p* value less than 0.05 were considered significant risk factors of schistosomiasis. Prior to assessing the multivariate analysis results, the generated model was tested for goodness of fit using the Hosmer–Lemeshow test and an omnibus test.

#### 2.4.2. Sequence Analysis

The obtained nucleotide sequences were subjected to a blast analysis on the NCBI website (https://blast.ncbi.nlm.nih.gov/Blast.cgi). to verify the specie of *Schistosoma* obtained. This was then followed by assembling and editing of raw sequences using the ATGC plug-in Genetyx ver. 12. Reference sequences were downloaded from GenBank and together with the obtained sequences, a multiple sequence alignment was constructed using clustalW1.6 [19]. The resultant fasta file of the multiple alignments was then converted to a mega file format and then utilized to construct a maximum likelihood phylogenetic tree based on the Kimura 2-parameter method and 1000 bootstrap replicates as a measure of the confidence interval in MEGA ver. 6 [20]. All the generated sequences in this study have been deposited in the DNA Data Bank of Japan and with accession numbers LC726150-LC726168.

## 3. Results

### 3.1. Demographic Characteristics of the Study Participants

A total of 421 school children from Lusaka and Siavonga districts were recruited. Of the participants, 55.1% (232/421) (95% CI: 50.21–59.91) were males, while 44.9% (189/421) (95% CI: 40.09–49.79) were females. The majority of the participants—85.3% (359/421) (95% CI: 81.44–88.45)—belonged to the age group 11–15 years. The age group of 16 years and above had the least number of participants, at 7.1% (30/421) (95% CI: 4.94–10.13) (Table 1).

### 3.2. Prevalence of Schistosomiasis Based on Microscopic Examination

The overall prevalence of schistosomiasis for the Lusaka and Siavonga districts was 9.7% (41/421) (95% CI: 7.16–13.08). The prevalence for Lusaka only was 5.7% (24/421) (95% CI: 3.76–8.48), while that of Siavonga was 4.0% (17/421) (95%CI: 2.44–6.51). Male participants had a higher prevalence at 6.2% (26/421) (95% CI: 4.15–9.03), compared to female participants—3.5% (15/421) (95% CI: 2.01–5.94). The age group of 11–15 years had the highest overall prevalence at 8.3% (35/421) (5.94–11.48) (Table 2).

The sex-specific prevalence was higher in male participants at 11.2% (26/232) (95% CI: 7.58–16.16) than female participants at 7.9% (15/189) (95% CI: 4.67–12.99), at *p*—0.038. The prevalence for Lusaka district was 11.9% (24/201) (95% CI: 7.95–17.43) while that of Siavonga was 7.7% (17/220) (95%CI: 4.70–12.29). The age group ≤10 years recorded the highest age-specific prevalence of schistosomiasis at 12.5% (4/32) (95%CI: 4.08–29.93) (Table 3).

### 3.3. Distribution of Potential Risk Factors

The study found that 59.4% (250/421) (95% CI: 54.51–64.08) of participants were involved in swimming and 51.5% (217/421) fished and (261/421) (95% CI: 57.15–66.62) bathed in permanent water bodies. Further, 89.1% (385/421) (95% CI: 85.60–91.81) had knowledge about schistosomiasis. The other potential risk factors are detailed in (Table 4).

### 3.4. Analysis of the Association between Potential Risk Factors and Schistosoma Positivity

In the bivariate analysis, 67% (6/9) of variables had an influence on *Schistosoma* positivity. The variables were swimming (*X*^2^ = 31.07, *p* < 0.001), fishing (*X*^2^ = 24.39, *p* < 0.001), sanitation (*X*^2^ = 26.68, *p* < 0.001), distance to water bodies (*X*^2^ = 26.60, *p* < 0.001), macroscopic results (*X*^2^ = 44.22, *p* = 0.001) and knowledge about schistosomiasis (*X*^2^ = 20.16, *p* < 0.001).

### 3.5. Maximum Likelihood Estimates of Risk Factors for Schistosomiasis

A stepwise binary logistic regression model was used to determine variables that could be predisposing risk factors for *schistosomiasis*. Variables with a *p*-value less than 0.25 in the bivariate analysis were included in the model. The test had an insignificant Hosmer–Lemeshow goodness-of-fit statistic (*p* = 0.916) and the omnibus test of model coefficients value of (*p* < 0.000) was obtained, indicating goodness-of-fit of the generated model. The risk factors were significant at *p*-value < 0.05. After adjustment for other variables in the model, three variables had a significant adjusted odds ratio (aOR) for schistosomiasis positivity and these were fishing, macroscopic results and distance to water bodies (Table 5). Participants that did not go fishing were (aOR: 0.008 (95% CI: 0.001–0.071)) times less likely to be positive for schistosomiasis than those that went fishing from the water bodies at (*p* = 0.001). Participants whose urine was not clear on macroscopy were (aOR: 9.98 (95% CI: 3.222–30.937)) times more likely to be positive for schistosomiasis than those whose urine was clear and participants that lived close to the water bodies were (aOR: 11.66 (95% CI: 3.290–41.310)) times more likely to have urogenital schistosomiasis than those that lived far from the water bodies (Table 5).

### 3.6. Phylogenetic Tree Analysis of S. haematobium nuclear ITS gene

**Figure 2:**
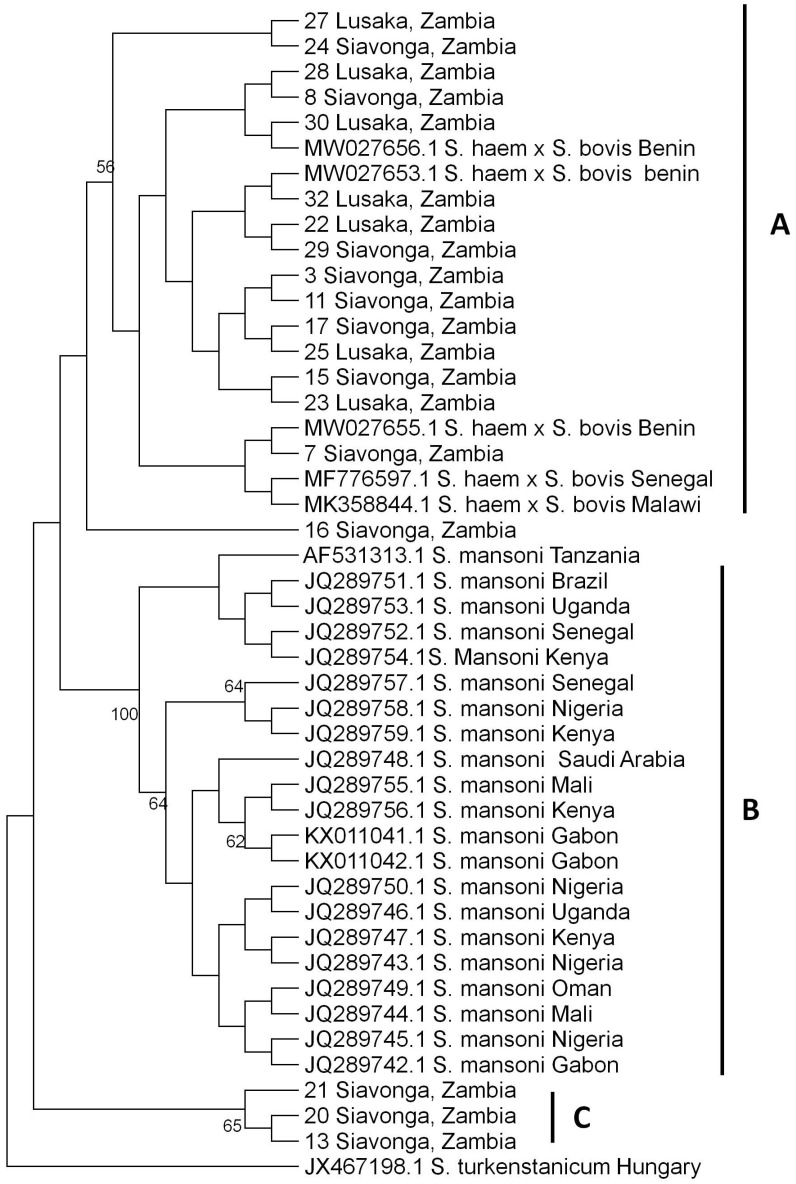
*Schistosoma haematobium* ITS maximum likelihood phylogenetic tree. The tree was generated from 981 bp nucleotide sequences using MEGA ver. 6 with 1000 bootstrap replicates as a confidence level.

The current study sequenced 19 samples of *S. haematobium* isolated from the urine samples of children using the nuclear ITS gene. The phylogenetic analysis of the *S. haematobium* ITS gene showed three main clusters, namely; A, B and C (Figure 2). Cluster A comprised of hybrids of *S. haematobium* and *S. bovis* species, cluster B comprised of *S. mansoni* and cluster C comprised of Zambian sequences of unknown species. All the sequences under study clustered in clusters A and C. The samples from Lusaka (*n* = 7) and Siavonga (*n* = 9) clustered in cluster A and fell in the same cluster with hybrids from Malawi, Senegal and Benin. Other samples from Siavonga (*n* = 3) clustered in cluster C and were unknown species (Figure 2).

## 4. Discussion

The current study investigated the prevalence and risk factors associated with schistosomiasis in school children in the Lusaka and Siavonga districts in Zambia, and also sequenced the *S. haematobium* nuclear ITS gene. The study reports an overall prevalence of 9.7% while the district-specific prevalence rates were 7.7% and 11.9% for Siavonga and Lusaka, respectively, in school-aged children. This prevalence, despite being low, indicates that urinary schistosomiasis is still endemic in the two districts of Zambia. Similar studies conducted among school children in the Kalabo, Serenje and Lusaka districts in Zambia reported prevalences of 1.4% (1/69), 3% (3/100), 13.1% (34/260) and 20.72% (328/1583), respectively [8,9,21]. The results for the current study are in between the three previously reported prevalences confirming that the disease is endemic in Zambia. Another study conducted on the prevalence of *S. haematobium* in Siavonga reported a slightly lower prevalence of 6.8% (192/2829). However, the above study was conducted among adult populations [17]. Nevertheless, our current results reflect more on the extent of the disease among school-aged children which is of special public concern. These observations imply that the current efforts toward prevention and control of the disease among children are not so effective. Kabuyaya et al. [22] attributed such observations to the use of a single dose of praziquantel during mass drug administration (MDA), a method that has been widely used in various parts of Zambia to combat the disease, which proved ineffective in the control and elimination of the disease. This is because reinfection may occur as people revert to their daily activities involving contact with water infested with the snail intermediate hosts even after being successfully treated with praziquantel [10,23]. Therefore, repeated standard doses accompanied by epidemiology monitoring are required to keep the infection at low transmission levels [23]. The observations on reinfection with schistosomiasis after successful treatment with praziquantel agree with the findings in the current study in which previous infection with schistosomiasis was reported in some participants that were previously treated with praziquantel successfully. Similar findings in studies conducted on school-aged children were reported in other parts of Africa in which prevalence rates of 10.2%, 12.9% and 14% were determined in Gambia, Sudan and Cote d’Ivoire, respectively [24,25,26].

Several previous studies have shown that the prevalence of schistosomiasis is higher in males than in females [14,24,26,27]. Similarly, our overall and sex-specific prevalences were higher in male school children than in female school children. The high prevalence in males could be attributed to their daily activities, as male children are more likely to be involved in water contact activities such as playing, swimming and fishing in freshwater bodies than females. Other activities such as watering livestock and other domesticated animals are also among factors that could predispose male children to contracting the disease [24,26]. On the contrary, the results of the present study, disagree with studies conducted in Nigeria, in which the highest prevalence of schistosomiasis was observed in females as opposed to males [5,28,29]. This was attributed to females being more involved in most chores such as washing dishes and fetching water from streams, which caused them to be in more contact with water than their male counterparts. Other studies conducted in Sudan and Cote d’Ivoire, however, found that boys and girls were equally infected with *S. haematobium* [24,25] and this was attributed to them being equally involved in everyday activities such as fetching water.

The results of the current study indicate that the highest overall prevalence of urinary schistosomiasis was recorded in the age group between 11 and 15 years. This finding is consistent with previous findings in studies conducted in Zambia in which the highest burden of disease was significantly high in the age groups of 10–15 years and 7–15 years [9,16]. The results obtained in the present study also corroborate many findings from previous studies conducted in other parts of Africa [5,14,24,26,27,30]. The high burden of disease in this age group is because children in this age group are mostly very active and have the highest contact levels with water which predispose them to *Schistosoma* infections. In most cases, children that harbour this infection are asymptomatic but complications may arise later as the adult worms and eggs migrate through the body resulting in haematuria, dysuria, anaemia, as well as lesions of the bladder that can cause increased risk of urinary bladder cancer [5]. Another finding worth noting from the current study is the high age-effect prevalence in children aged ≤ 10 years. This observation was also reported in Malawi where pre-school aged children were found to be infected with *S. haematobium* [31]. This was because children accompanied their mothers when conducting water contact activities such as water fetching and washing [32]. This is an important factor that may exacerbate transmission activities in young children [31,32].

Further, it was observed that the prevalence of *S. haematobium* among the children in the schools studied in Siavonga varied despite having similar environmental and social–economic characteristics. The highest school-specific prevalence was recorded at Siamatika followed by Butete, while zero prevalence rate of the disease was recorded at Chininde School. The differences in the prevalence rates could be attributed to presence of different populations of the intermediate host snails with differences in cercarial shedding patterns and also differences in water contact behaviours by human hosts which may lead to variations in exposure to cercariae-contaminated water [4,33]. These differences have also been documented by previous studies conducted in Zambia and Burkina Faso [4,34,35]. Ndassi et al. [35], also attributed such findings to differences in the number of water bodies present in the area, proximity to streams and a lack of health education and poor hygiene in some communities. The current study observed that the appearance of urine on macroscopy or physical inspection had a positive linear association with schistosomiasis, similar to a study in Gambia that reported that visible blood in urine (macrohaematuria) and microhaematuria were associated with the presence of *S. haematobium* in urine [26]. Similarly, studies conducted in Nigeria and Burkina Faso found a positive correlation between macrohaematuria and the presence of *S. haematobium* eggs in urine [34,36]. The findings in these studies corroborate findings in the present study in which the appearance of urine on macroscopy was positively associated with schistosomiasis. This has become an increasingly common association and has been demonstrated as a reliable diagnostic method for the detection of *S. haematobium* and is a good indicator of infection. In fact, it is an early defining indicator of infection even if not all presence of blood in urine is associated with infection [26,34].

Several studies have found a significant association between schistosomiasis infection and activities such as bathing, fishing, swimming, playing and fetching water in the rivers and other water bodies [37,38,39,40]. Other factors reported to have had a positive relationship with schistosomiasis infection include knowledge about the snail host, poor sanitation, proximity to water bodies and history of urogenital schistosomiasis [37,38,39,40,41,42]. The findings in these studies are similar to the current results where a positive relationship between factors such as prior infection, distance to water bodies, fishing, swimming, poor sanitation and knowledge about the disease and schistosomiasis was observed. Furthermore, the present results indicate that having cloudy or bloody urine and proximity to water bodies were risk factors of schistosomiasis while fishing was a protective factor in which children who did not go fishing were less likely to test positive for schistosomiasis than those that did. Our observations corroborate results from a study conducted in Nigeria in which the odds ratios observed among children who played/bathed, fetched water and swam, indicated that the more the children visited the cercariae-infested water bodies, the higher the chances of them becoming infected compared to those that did not [38]. However, the present study observations contradict studies conducted in Nigeria and Gambia in which no significant association was found between schistosomiasis and water contact activities, and between schistosomiasis and other factors such as knowledge about the disease [5,26,43].

A phylogenetic tree analysis is used to determine evolutionary relationships among a set of organisms or groups of organisms. This can therefore provide insight on the molecular epidemiology of various organisms from different study areas [38]. In the present study, the phylogenetic tree analysis of *S. haematobium* ITS gene indicates that some isolates (*n* = 9) of *S. haematobium* from Siavonga and Lusaka (*n* = 7) were closely related to hybrid species of *S. haematobium* × *S. bovis* from Benin, Senegal and Malawi. This implies that the *S. haematobium* isolated from the two study areas in Zambia is mainly of one strain; hybrids of *S. haematobium* × *S. bovis*. Therefore, these results indicate the possibility of hybridization of *S. haematobium*, the causative agent of urogenital schistosomiasis in Zambia. Hybrids of *S. haematobium* × *S. bovis* have been reported in humans and intermediate snail hosts in several studies conducted in most western Africa countries such as Mali, Senegal, Niger, Côte d’Ivoire, Kenya and currently in Malawi and Corsica, France [15,44,45,46,47]. *Schistosoma bovis* is a parasite of mostly ruminants and also rodents [48]. The two species, *S. bovis* and *S. haematobium*, are closely related, have morphologically similar cercariae and may be found in the same fresh water bodies [49]. The hybridization between human-specific (*S. haematobium*) and animal-specific *Schistosoma* species (*S. bovis*) is the most common form of hybridization and raises the possibility of zoonotic parasite strain transmission [45]. This occurs as animals and humans frequent the same water sources leading to the interplay of the species they carry [50]. Hybridization might influence disease transmission and alter phenotypic characteristics of parasites in both human and animal hosts. Moreover, laboratory hybrids have shown to exhibit increased virulence, expanded intermediate snail host spectrum, maturation and egg production. Further, hybrids of the parasite also increase genetic diversity and population structure [44,45,50]. However, not much is known about the biological and pathological consequences or zoonotic characteristics of this infectio. Therefore, there is a need to study hybrids of schistosome parasites and to determine how such hybrids might influence the epidemiology and control of schistosomiasis in terms of virulence, zoonotic potential and resistance to treatment [25,45].

Our study, to the best of our knowledge, is the first to sequence and identify *S. haematobium* strains isolated in Zambia and therefore provides cardinal information to public health officials. It also serves as a basis for more molecular work on urinary schistosomiasis to determine the genetic diversity of its causative agent as this will aid in understanding the molecular epidemiology of the disease in Zambia.

## 5. Conclusions

The findings of the current study indicate that *S. haematobium* infection is still endemic in rural Siavonga and peri-urban areas of Lusaka, Zambia and is associated with several risk factors related to water contact activities. The *S. haematobium* isolated from the current study was closely related to hybrids of *S. haematobium* × *S. bovis*, indicating the potential risk to public health. It is thus imperative to educate people on the treatment, prevention and control of the disease, especially in communities where high prevalence has been reported.

## Figures and Tables

**Table 1:** Socio-demographic characteristics of study participants.

Demographic Characteristics	Category	Frequency (*n* = 421)	Proportion (%)	95% CI
**Gender**	Male	232	55.1	50.21–59.91
	Female	189	44.9	40.09–49.79
**Age (years)**	≤10	32	7.6	5.34–10.67
	11–15	359	85.3	81.44–88.45
	≥16	30	7.1	4.94–10.13
**School Grade**	1–4	42	10.0	7.36–13.34
	5–7	379	90.0	86.66–92.64
**School**	Nabutezi	47	11.2	8.93–14.66
	Chininde	46	10.9	8.18–14.40
	Mpango	44	10.5	7.77–13.87
	Siamatika	42	10.0	7.36–13.34
	Butete	41	9.7	7.16–13.08
	Ng’ombe	201	47.7	42.90–52.63

*n* = number of respondents; % = Percentage; CI = Confidence interval.

**Table 2:** Prevalence of schistosomiasis across sex, age, schools and school grades.

Character	Categories	Frequency (*n* = 421)	Prevalence	95% CI
**District**	Lusaka	24	5.7	3.76–8.48
	Siavonga	17	4.0	2.44–6.51
	Overall	41	9.7	7.16–13.08
**Sex**	Male	26	6.2.	4.15–9.03
	Female	15	3.5	2.01–5.94
**Age**	≤10	4	0.9	0.30–2.58
	11–15	35	8.3	5.94–11.48
	≥16	2	0.5	0.08–1.89
**School**	Nabutezi	2	0.5	0.08–1.89
	Chininde	4	0.9	0.30–2.58
	Mpango	3	0.7	0.18–2.25
	Siamatika	5	1.2	0.44–2.91
	Butete	3	0.7	0.18–2.25
	Ng’ombe	24	5.7	3.76–8.48
**Grade**	1–4	3	0.7	0.18–2.25
	5–7	38	9.0	6.54–12.28

*n* = number of respondents; % = Percentage; CI = Confidence interval.

**Table 3:** District, Age, School, Grade and Sex-Specific Prevalences.

Character	Categories	Frequency	*n*	Prevalence	95% CI
**District**	Lusaka	24	201	11.9	7.95–17.43
	Siavonga	17	220	7.7	4.70–12.29
**Sex**	Male	26	232	11.2	7.58–16.16
	Female	15	189	7.9	4.67–12.99
**Age**	≤10	4	32	12.5	4.08–29.93
	11–15	35	359	9.7	6.97–13.41
	≥16	2	30	6.7	1.16–25.51
**School**	Nabutezi	2	47	4.3	0.74–15.73
	Chininde	4	46	8.7	2.82–21.69
	Mpango	3	44	6.8	1.78–19.71
	Siamatika	5	42	11.9	4.47–26.43
	Butete	3	41	7.3	1.91–21.01
	Ng’ombe	24	201	11.9	7.95–17.43
**Grade**	1–4	3	42	7.1	1.86–20.55
	5–7	38	379	10.0	7.28–13.61

*n* = number of respondents; % = Percentage; CI = Confidence Interval.

**Table 4:** Proportion of potential risk factors among respondents.

Potential Risk Factors	Category	Frequency (*n* = 421)	Proportion (%)	95% CI
**Swimming**	Yes	250	59.4	54.51–64.08
	No	171	40.6	35.92–45.49
**Bathing**	Yes	217	51.5	46.66–56.40
	No	204	48.5	43.60–53.34
**Fishing**	Yes	261	62.0	57.15–66.62
	No	160	38.0	33.38–42.85
**Washing/water fetching**	Yes	279	66.3	61.50–70.74
	No	142	33.7	29.26–38.50
**Macroscopic results**	Clear/Amber	327	77.7	73.33–81.50
	Cloudy/Bloody	94	23.3	18.50–26.67
**Prior treatment**	No	155	36.8	32.23–41.64
	Yes	226	53.7	48.79–58.51
**Distance to water**	Far (>5 km)	365	86.7	82.99–89.72
	Near (<5 km)	56	13.3	10.28–17.01
**Knowledge about disease**	Yes	375	89.1	85.60–91.81
	No	46	10.9	8.19–14.40
**Herding livestock**	Yes	230	54.6	49.74–59.44
	No	191	45.4	40.56–50.26
**Sanitation**	Good	360	85.5	81.70–88.66
	Poor	61	14.5	11.34–18.30
**Prior infection**	Yes	173	41.1	36.38–45.97
	No	248	58.9	54.03–53.62

*n* = number of respondents; % = Percentage; CI = Confidence interval.

**Table 5:** Risk factors for Schistosomiasis.

Variable	Level	aOR	95% CI	*p*-Value
Fishing (*n* = 421)	Yes	Ref	-	-
	No	0.008	0.001–0.071	0.001 **
Macroscopic results (*n* = 421)	Clear	Ref	-	-
	Not clear	9.98	3.222–30.937	0.001 **
Distance to water bodies (*n* = 421)	Far	Ref	-	-
	Near	11.66	3.290–41.310	0.001 **

** = Significant at 0.05; *n* = Number of Children; CI = Confidence interval, Significant level < 0.05; aOR = Odds ratio.

## Data Availability

All the data has been provided in this article and any questions regarding the data that has been presented in the present study can be addressed to the corresponding author. Sequences for the *S. haematobium* ITS gene have been deposited in the DNA Data Bank of Japan (DDBJ) with accession numbers LC726150-LC726168.
